# Supplementary material for: Defining key roles for auxiliary proteins in an ABC transporter that maintains bacterial outer membrane lipid asymmetry
Source: eLife. 2016 Aug 16;5:e19042. doi: 10.7554/eLife.19042 (PMC5016091; doi:10.7554/eLife.19042)

**Figure 5 – source data 1.** Source data for ATPase assay.

NADH utilization rates for coupled ATPase assays of indicated complexes

(NADH fluorescence vs time curves for each complex at indicated ATP concentrations; three technical replicates)


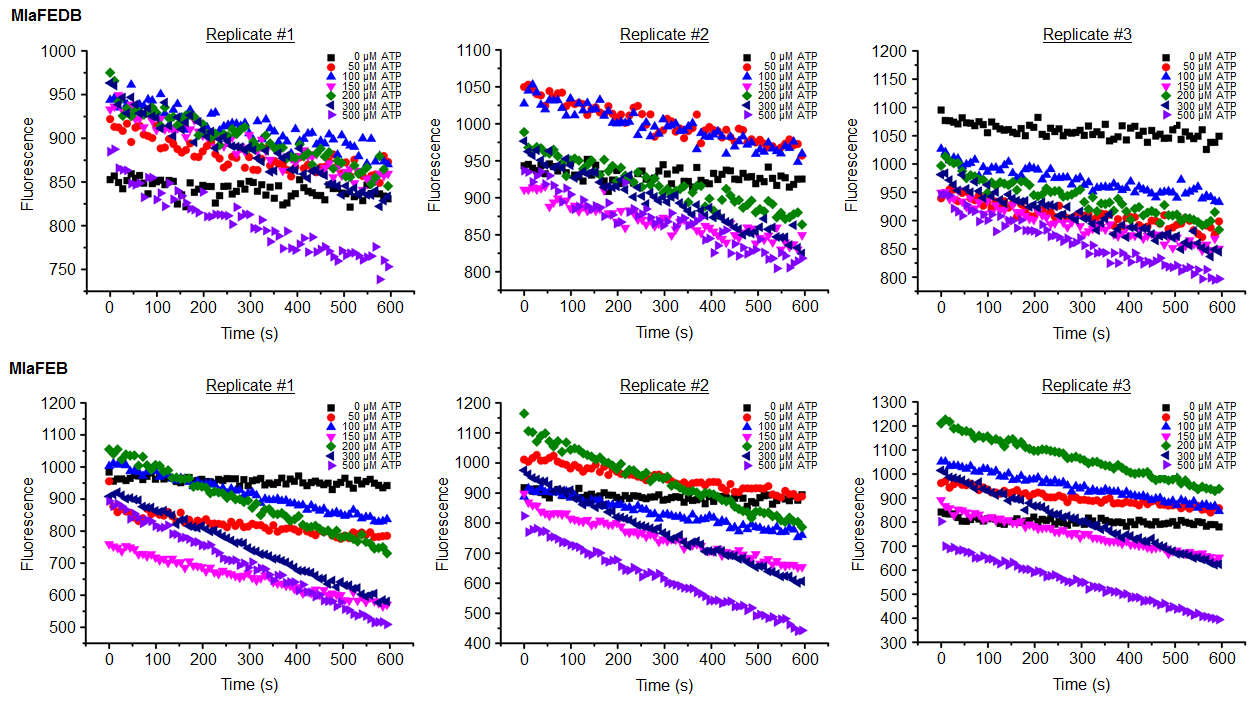


NADH utilization rates for coupled ATPase assays of indicated complexes

(NADH fluorescence vs time curves for each complex at indicated ATP concentrations; three technical replicates)


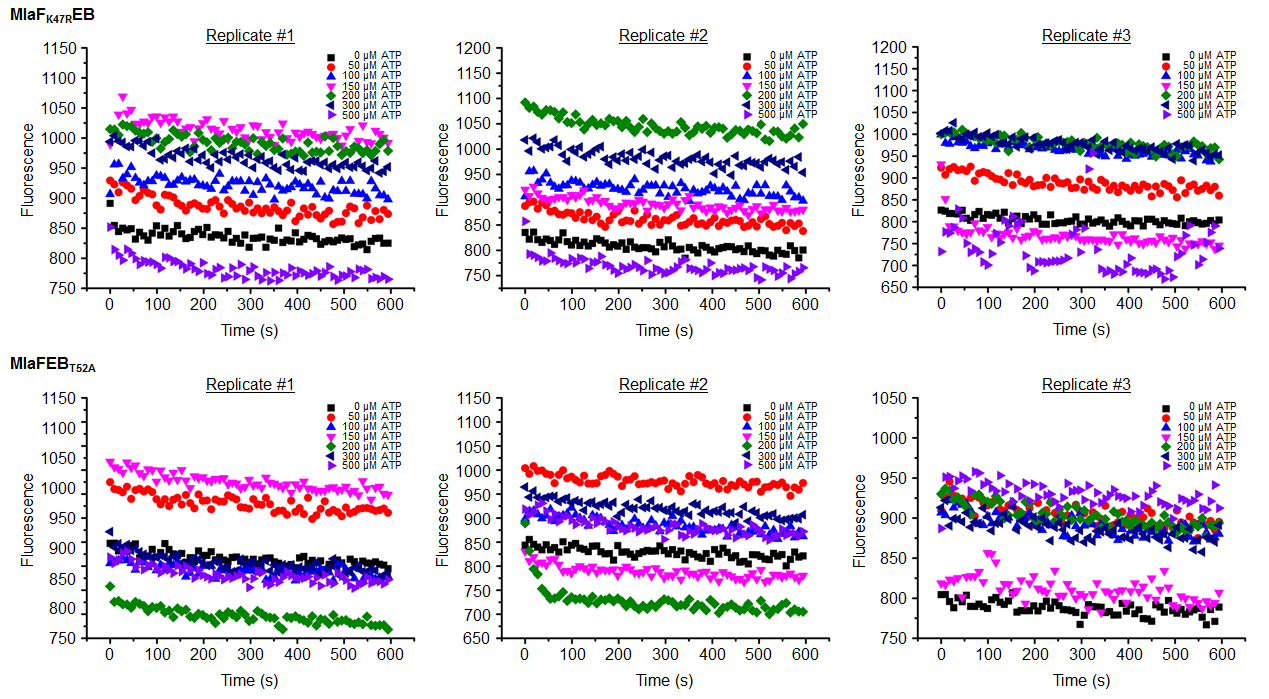


Table showing derivation of ATP hydrolysis rates (in red) plotted in Figure 5


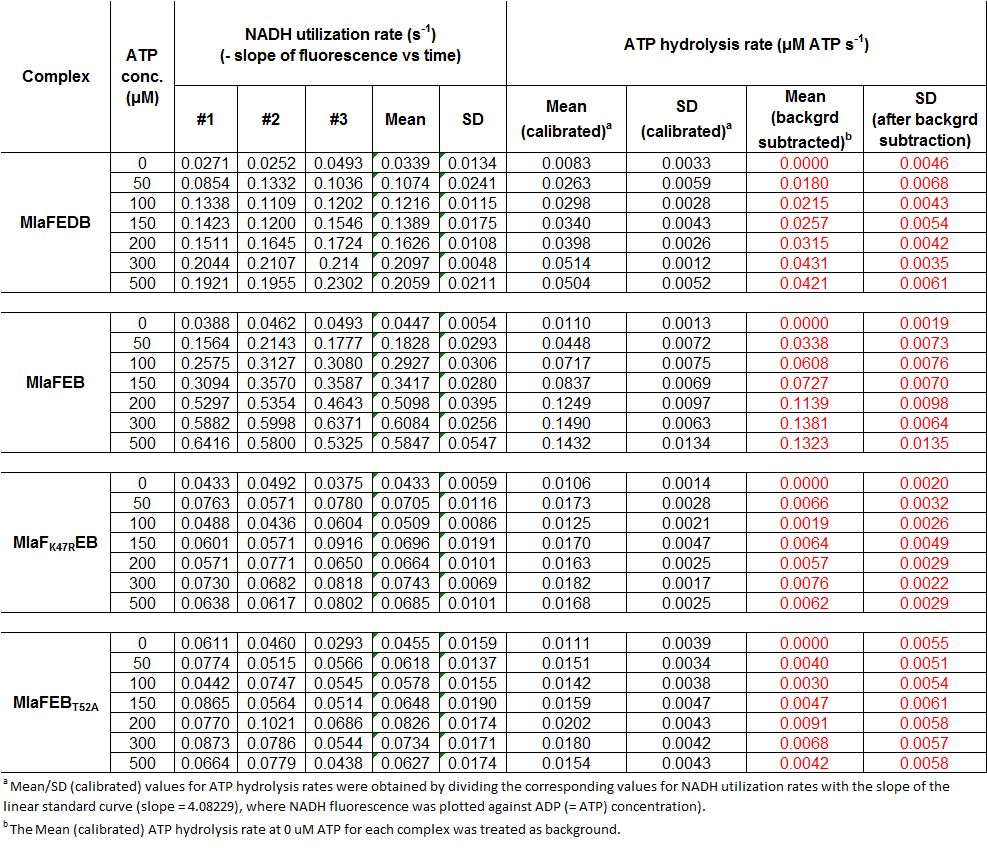

Supplement: Figure 5—source data 1. — DOI: http://dx.doi.org/10.7554/eLife.19042.016 [file elife-19042-fig5-data1.docx]
