# Supplementary material for: Defining key roles for auxiliary proteins in an ABC transporter that maintains bacterial outer membrane lipid asymmetry
Source: eLife. 2016 Aug 16;5:e19042. doi: 10.7554/eLife.19042 (PMC5016091; doi:10.7554/eLife.19042)
Supplement: Supplementary file 1. — DOI: http://dx.doi.org/10.7554/eLife.19042.018 [file elife-19042-supp1.docx]

**Supplementary File 1.** Bacteria strains used in this study.

| **Strains** | **Relevant genotypes or characteristics** | **Reference** |
| --- | --- | --- |
| MC4100 | *F^-^ araD139* Δ(*argF-lac*) *U169 rpsL150 relA1 flbB5301 ptsF25 deoC1 ptsF25 thi* | Lab collection |
| BW25113 | *F*^-^ ∆(*araD-araB*)*567* ∆*lacZ4787::rrnB-3 λ^-^ rph-1* ∆(*rhaD-rhaB*)*568 hsdR514* |  |
| Novablue | *endA1 hsdR17* (r_K12_^–^ m_K12_^+^) *supE44 thi-1 recA1 gyrA96 relA1 lac* F′ [*proA^+^B^+^lacI^q^Z*Δ*M15*::Tn*10*] | Novagen |
| BL21(λDE3) | *F^–^* *ompT* *gal* *dcm* *lon* *hsdS_B_*(r_B_^–^m_B_^–^) *λ(DE3* [*lacI* *lacUV5*-*T7 gene 1* *ind1* *sam7* *nin5*]) [*malB*^+^]_K-12_(λ^S^) | Novagen |
| JW3160 | BW25113 ∆*mlaD::kan* | (Baba et al., 2006) |
| JW3161 | BW25113 ∆*mlaE::kan* | (Baba et al., 2006) |
| JW3162 | BW25113 ∆*mlaF::kan* | (Baba et al., 2006) |
| TSH001 | BW25113 ∆*mlaB::cam* | This study |
| CZS012 | MC4100 ∆*mlaD::kan* | This study |
| CZS013 | MC4100 ∆*mlaE::kan* | This study |
| CZS014 | MC4100 ∆*mlaF::kan* | This study |
| TSH002 | MC4100 ∆*mlaB::cam* | This study |
| EH150 | *psd-2_(ts)_* *purA*^+^ | (Hawrot et al., 1975) |
