# Supplementary material for: Defining key roles for auxiliary proteins in an ABC transporter that maintains bacterial outer membrane lipid asymmetry
Source: eLife. 2016 Aug 16;5:e19042. doi: 10.7554/eLife.19042 (PMC5016091; doi:10.7554/eLife.19042)
Supplement: Supplementary file 2. — DOI: http://dx.doi.org/10.7554/eLife.19042.019 [file elife-19042-supp2.docx]

| **Supplementary File 2.** Plasmids used in this study. | | | | |
| --- | --- | --- | --- | --- |
| **Plasmids** | **Relevant genotypes or characteristics** | **Plasmid construction** | | **References** |
|  |  | **PCR template^a^** | **PCR primers^b^** |  |
| pET22b(+) | P*_T7_lacO* inducible expression vector, contains N-terminal PelB signal peptide for periplasmic localization; Amp^R^ | - | - | Novagen |
| pET23/42 | P*_T7_* inducible expression vector, contains multiple cloning site of pET42a(+) in pET23a(+) backbone, C-terminal His_8_-tag; Amp^R^ | - | - | (Wu et al., 2006) |
| pET23/42(N) | P*_T7_* inducible expression vector, contains the first multiple cloning site of pETDuet-1 in pET23a(+) backbone, N-terminal His_6_-tag; Amp^R^ | - | See Materials and Methods | This study |
| pET22/42 | P*_T7_lacO* inducible expression vector, contains multiple cloning site of pET42a(+) in pET22b(+) backbone; Amp^R^ | - | - | (Chng et al., 2010b) |
| pCDF Duet-1 | P*_T7_lacO-*dependent dual expression vector; Sm^R^, Spec^R^ | - | - | Novagen |
| pET23/42*mlaF-His* | Encodes full length MlaF with C-terminal His_8_ tag; Amp^R^ (p*mlaF-His*) | ch. DNA | MlaF-N-NdeI FWD MlaF-C-XhoI REV | This study |
| pET23/42*His-mlaE* | Encodes full length MlaE with N-terminal His_6_ tag; Amp^R^ (p*His-mlaE*) | ch. DNA | MlaE-N-EcoRI FWD MlaE-C-AvrII REV | This study |
| pET23/42*mlaD-His* | Encodes full length MlaD with C-terminal His_8_ tag; Amp^R^ (p*mlaD-His*) | ch. DNA | MlaD-N-NdeI FWD MlaD-C-XhoI REV | This study |
| pET23/42*His-mlaB* | Encodes full length MlaB with N-terminal His_6_ tag; Amp^R^ (p*His-mlaB*) | ch. DNA | MlaB-N-EcoRI FWD MlaB-C-AvrII REV | This study |
| pET22/42*mlaF(His-E)DCB* | Encodes full length MlaF, MlaE, MlaD, MlaC and MlaB with N-terminal His_6_ tag before MlaE; Amp^R^ | ch. DNA | See Materials and Methods | This study |
| **Plasmids** | **Relevant genotypes or characteristics** | **Plasmid construction** | | **References** |
|  |  | **PCR template^a^** | **PCR primers^b^** |  |
| pET22/42*mlaF(His-E)D* | Encodes full length MlaF, MlaE and MlaD with N-terminal His_6_ tag before MlaE; Amp^R^ | pET22/42*mlaF(His-E)DCB* | MlaF-N-NdeI FWD MlaD-C-AvrII REV | This study |
| pET22/42*mlaF(His-E)* | Encodes full length MlaF and MlaE with N-terminal His_6_ tag before MlaE; Amp^R^ | pET22/42*mlaF(His-E)DCB* | MlaF-N-NdeI FWD MlaE-C-AvrII REV | This study |
| pET22/42*mlaF_K47R_(His-E)* | Encodes full length MlaF_K47R_ and MlaE with N-terminal His_6_ tag before MlaE; Amp^R^ | pET22/42*mlaF(His-E)* | MlaF-K47R-N FWD MlaF-K47R-C REV | This study |
| pET22/42*mlaF-His* | Encodes full length MlaF with C-terminal His_8_ tag; Amp^R^ | ch. DNA | MlaF-N-NdeI FWD MlaF-C-XhoI REV | This study |
| pET22/42*mlaF_K47R_-His* | Encodes full length MlaF_K47R_ with C-terminal His_8_ tag; Amp^R^ | pET22/42*mlaF-His* | MlaF-K47R-N FWD MlaF-K47R-C REV | This study |
| pCDF*mlaB* | Encodes full length MlaB (replaces both cloning sites); Sm^R^, Spec^R^ | ch. DNA | MlaB-N-NcoI FWD MlaB-C-XhoI REV | This study |
| pCDF*mlaB_T52A_* | Encodes full length MlaB_T52A_ (replaces both cloning sites); Sm^R^, Spec^R^ | pCDF*mlaB* | MlaB-T52A-N FWD MlaB-T52A-C REV | This study |
| pET22/42s*mlaD-His* | Encodes soluble domain of MlaD (a.a. 29-183) with C-terminal His_8_ tag; Amp^R^ | ch. DNA | MlaD-SD-N-NdeI-FWD MlaD-SD-C-XhoI-REV | This study |
| pET22b*lolB-His* | Encodes LolB (a.a. 23-207) with a C-terminal His_6_ tag and the *pelB* signal peptide; Amp^R^ | - | - | (Chng et al., 2010b) |

^a^ ch. DNA = MC4100 chromosomal DNA

^b^ primer sequences are listed in Supplementary File 3.
