# Supplementary material for: Defining key roles for auxiliary proteins in an ABC transporter that maintains bacterial outer membrane lipid asymmetry
Source: eLife. 2016 Aug 16;5:e19042. doi: 10.7554/eLife.19042 (PMC5016091; doi:10.7554/eLife.19042)
Supplement: Supplementary file 3. — DOI: http://dx.doi.org/10.7554/eLife.19042.020 [file elife-19042-supp3.docx]

**Supplementary File 3.** Primers used in this study.

| **Primers** | **Sequence (5’ to 3’)**^a^ |
| --- | --- |
| MlaF-N-NdeI FWD  MlaF-C-XhoI REV  MlaE-N-EcoRI FWD  MlaE-C-AvrII REV  MlaD-N-NdeI FWD  MlaD-C-XhoI REV  MlaD-C-AvrII REV  MlaD-SD-N-NdeI-FWD  MlaD-SD-C-XhoI-REV  MlaB-N-EcoRI FWD  MlaB-N-NcoI FWD  MlaB-C-XhoI REV  MlaB-C-AvrII REV  1-Linker-NHis-MlaE-N FWD  1-Linker-NHis-MlaE-C REV  2-Linker-NHis-MlaE-N FWD  2-Linker-NHis-MlaE-C REV  MlaF-K47R-N FWD  MlaF-K47R-C REV  MlaB-T52A-N FWD  MlaB-T52A-C REV  MlaB-cam-N5  MlaB-cam-C3 | ATGCCATATGGAGCAGTCTGTGGC  ATGCCTCGAGACTCCCTGGTAAAAGATCAGC  ATGCGAATTCGATGCTGTTAAATGCGCTGGC  ATGCCCTAGGTCAATTCCCAAACATCAATGCGG  ATGCCATATGCAAACGAAAAAAAATGAAATTTGGG  ATGCCTCGAGTTTCGTACCCACAGGTTC  ATATCCTAGGTTATTTCGTTGTACCCACAGGTTC  ATCACATATGGCGAACGTGACGTCC  ATCTCTCGAGTTTCGTTGTACCCACAG  ATGCGAATTCAGCGAGTCACTGAGCTG  ATATCCATGGGCAGCGAGTCACT  ATATCTCGAGTTAACGAGGCAGAACATCAGC  ATGCCCTAGGTTAACGAGGCAGAACATCAGC  CACCACAGCCAGGATCCGAATTCGATGCTGTTAAATGCGCTG  CTTTTACCAGGGAGTTAAGCCACTCCATCACCATCATCACCACAGCCAG  ATGGGCAGCAGCCATCACCATCATCACCACAGC  CTTTTACCAGGGAGTTAAGCCACTCATGGGCAGCAGC  ATCGGGCATCGGTCGTACGACGCTACTCC  GGAGTAGCGTCGTACGACCGATGCCCGAT  CGCGTGGATGCGGGGGGACTG  CAGTCCCCCCGCATCCACGCG  TTTCTCAACAGAAAATCACTCTGGAAGAGAAAAAATAATG GTGTAGGCTGGAGCTGCTTC  TCCGGCCTGAAAAAATTTAACGAGGCAGAACATCAGCAGGCATATGAATATCCTCCTTAG |

^a^Relevant restriction sites are underlined.
